# Public perception, knowledge and factors influencing COVID-19 vaccine acceptability

**DOI:** 10.4314/gmj.v57i3.14

**Published:** 2023-09

**Authors:** Amnuay Kleebayoon, Viroj Wiwanitkit

**Affiliations:** D Y Patil University, Navi Mumbai, Maharashtra, India

Dear Editor-in-Chief,

We would like to share ideas on the publication “A survey of public perception, knowledge and factors influencing COVID-19 vaccine acceptability in five communities in Ghana”.^1^ The study selected families in five Accra areas using a modified cluster-sampling method. This sampling strategy may create selection bias and limit the findings' generalizability to the Ghanaian population. The chosen localities may not be typical of the country, potentially impacting the study's external validity. The survey has 997 participants, which may not be enough to provide an in-depth view of the public's perspective and knowledge of COVID-19 in Ghana. Furthermore, the study provides no information on the sample's representativeness in terms of demographic variables such as age, gender, and socioeconomic level. Without this information, determining whether the sample is genuinely representative of the population is difficult. To investigate parameters related to vaccine acceptance, the study used ordinary least square linear regression analysis. While this technique can detect connections, it should be noted that it cannot demonstrate causality. Without additional data, the report should be cautious in interpreting the results as causative correlations.

Concerns are voiced each time a new COVID-19 vaccination is developed and made available to the general population. The villagers might worry if they hear unpleasant news. The main source of uncertainty is the misunderstanding that the COVID-19 vaccination will almost certainly affect the broader public.^2^ The environment and the start of the COVID-19 outbreak influence resistance patterns.^3^ The effect of promotion may alter over time since the hesitant pattern changes with time. If additional study is required, it should focus on determining and resolving why people are reluctant to get vaccines, such as misleading information, mistrust, and access issues, and developing and assessing practical solutions to vaccine hesitancy in various situations.

## Authors' response

Thank you for the email and the ideas you shared. The authors have read the letter to the Editor and are grateful for the comments or ideas shared. The authors have responses to the issues raised in the letter, and these are outlined below:

The authors noted the small sample size and, therefore, a limitation to the generalizability of the findings. This was stated in the study limitation section of the article that the study was limited to areas in the Greater Accra region of Ghana. Whilst our study findings cannot be generalised to the whole Ghanaian population, they certainly provide useful information on the acceptability of COVID-19 vaccination by section of the Ghanaian public.

Information about participants' socio-demographic characteristics was presented in Table 1, which provides information on all the variables. Example: *Most respondents were males (57.6%) aged 25 years and below (36.2%). The age rangedfrom 18-86 years, with 30 years median age. Over one-quarter of the participants were unemployed*. In no part of the manuscript was it stated that the sample is “genuinely representative” of the Ghanaian population.

The study was cross-sectional, as indicated, so proving causality can be very difficult. Knowing this, the authors tested for associations and correlations of variables, which indicates that the values vary. It was never emphatically stated or implied in the article that our study findings were interpreted as “causative correlations”.

The ordinary least square regression model was only used to estimate the coefficients of linear regression equations, which describe the relationship between the independent and dependent variables. So, the ordinary least square regression model was not used to establish causality.
